# Preliminary data on the presence of an alternate vanadium nitrogenase in a culturable cyanobiont of *Azolla pinnata* R. Brown: Implications on Chronic Kidney Disease of an unknown etiology (CKDu)

**DOI:** 10.1016/j.dib.2018.11.073

**Published:** 2018-11-17

**Authors:** B.L.D.U. Pushpakumara, D. Gunawardana

**Affiliations:** Department of Botany, University of Sri Jayewardenepura, Sri Lanka

## Abstract

In a recent paper titled “How a taxonomically-ambiguous cyanobiont and vanadate assist in the phytoremediation of cadmium by *Azolla pinnata*: implications for CKDu” (Atugoda et al., 2018) [1] it was shown by us, that plant health and phytoremediation capacities, of *Azolla pinnata* R. Brown, were elevated in the presence of vanadate, a vanadium containing ion. This highlighted a possibility, that either the major or minor cyanobionts of *Azolla pinnata*, could possess a vanadium dependent nitrogenase enzyme, as an alternate nitrogenase, in addition to the molybdenum counterpart. In this data article, we report the isolation of a minor cyanobiont which we name as *Fischerella uthpalarensis.* We grew *Fischerella uthpalarensis*, exclusively in N-free media, with only molybdenum (Mo+ V-), with only vanadium (V+ Mo-) and with neither (negative control), to find out the growth patterns in the relevant media. While *F. uthpalarensis* grew as green colored consistencies, increasing gradually in turbidity, for 4 weeks in culture, both, in the presence of molybdenum (Mo+ V-), as well as vanadium (V+ Mo-), the negative control, showed no, or very little growth. This alludes to the presence of dual nitrogenases in *Fischerella uthpalarensis*. An attempt was also made by us to unravel the *vnf* genes, responsible for the V-nitrogenase. However, it was not possible to PCR amplify the *vnf* genes, from both, the unculturable major (using total DNA from the Azolla-*Nostoc azollae* symbiosis) and minor (DNA directly from the cultured *F. uthpalarensis*) cyanobionts. This is the first time, to our knowledge, that an endosymbiotic cyanobacterium inside a plant compartment, has been shown to contain two possible nitrogenase systems.

## Specifications table

**Table:** 

**Subject area**	*Plant-microbe interactions*
**More specific subject area**	*Symbiotic cyanobacteria in Azolla pinnata* R. Brown
**Type of data**	*Figures*
**How data was acquired**	*Optical microscope, and gel documentation system*
**Data format**	*Qualitative (Observation and Analysis)*
**Experimental factors**	*Separate culture media were used containing either molybdenum or vanadium for the growth of an isolated cyanobacterium from Azolla pinnata.*
**Experimental features**	*Culture growth was observed and microscopy was performed to assess the presence of heterocysts. The vnf genes were hunted for, using PCR.*
**Data source location**	*Colombo, Sri Lanka.*
**Data accessibility**	*Data is presented here as an open access resource*
**Related research article**	*Atugoda D.R.A.M.T.R, Mandakini L.L.U, Bandara N.J.G.J., Gunawardana, D. (2018) How a taxonomically-ambiguous cyanobiont and vanadate assist in the phytoremediation of cadmium by Azolla pinnata: implications for CKDu. Environment and Pollution 7(1) 53–65.*[1]

## Value of the data

• Dual nitrogenase systems are rare in biology, and therefore, the data we are presenting here, would be of immense value for researchers, who wish to characterize further, the alternate nitrogenase enzyme, and the genetic basis of this two-prong nitrogenase system, while from an applied sciences perspective, molybdenum being found at only 1–2 ppm in soils, while vanadium being present in concentrations several fold higher, measured to be ~ 100 ppm on average [2], suggests to us, that our data, can have ripple effects on many downstream applications.
• According to [3], during the rainy season in the dry zone of Sri Lanka, where rainwater is slightly acidic in nature, the acid-soluble fraction of vanadium in the soil, becomes heightened in bioavailability, leaching into sources of surface and ground water, which points to *Azolla pinnata* being a good candidate to remediate irrigated water in paddy fields, especially those in CKDu areas. Furthermore, *Azolla pinnata*, fed with vanadium, can also be employed in industrial wastewater remediation ponds, to sanitize heavy metals, from industrial effluents.
• Azolla is a widespread biofertilizer, used in irrigated and aerobic rice fields, in the floating form and as harvested green manure respectively, to replenish the nitrogen needs of irrigated and aerobic rices, of which, the utilization, can now be broadened and intensified, in vanadium rich soil.
• *Azolla pinnata* doubles its biomass in 5–6 days [4] and in vanadium enriched soils, the doubling of biomass and carbon fixation, can be rapid, while it is also worth gathering empirical evidences, whether *Azolla pinnata*, can be a sequestering sink, not just for carbon dioxide, but even other greenhouse gases.
• *Azolla pinnata* can furnish a wide spectrum of renewable biofuels, which are low cost, while having minimum maintenance costs, including ethanol, biogas, bio-oil and bio-hydrogen gas [4], which makes such quests to unearth the “biofuel” potential of Azolla, timely.

## 1 Data

**Fig. 1:**
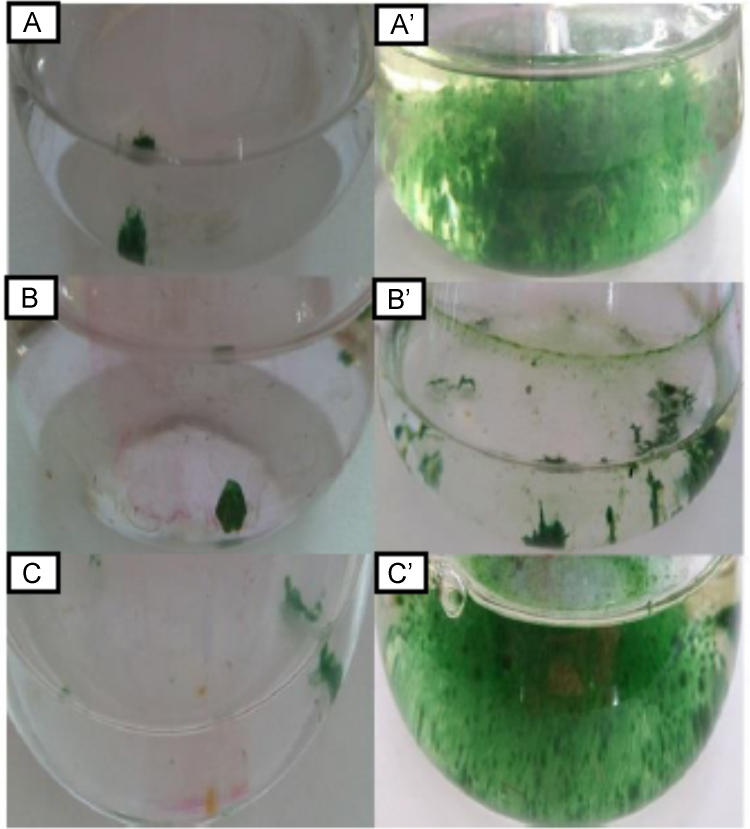
Flasks showing the growth of the isolated cyanobiont at time zero and four weeks of incubation; A–C – Flasks with V+Mo-, V- Mo- and V-Mo+ respectively at time zero; A’–C’- Flasks with V+Mo-, V- Mo-and V-Mo+ respectively after four weeks of incubation. V+Mo-, V- Mo- and V-Mo+ represent BG11_0_ medium with V (without Mo), without both V and Mo and with Mo (without V), respectively.

**Fig. 2:**
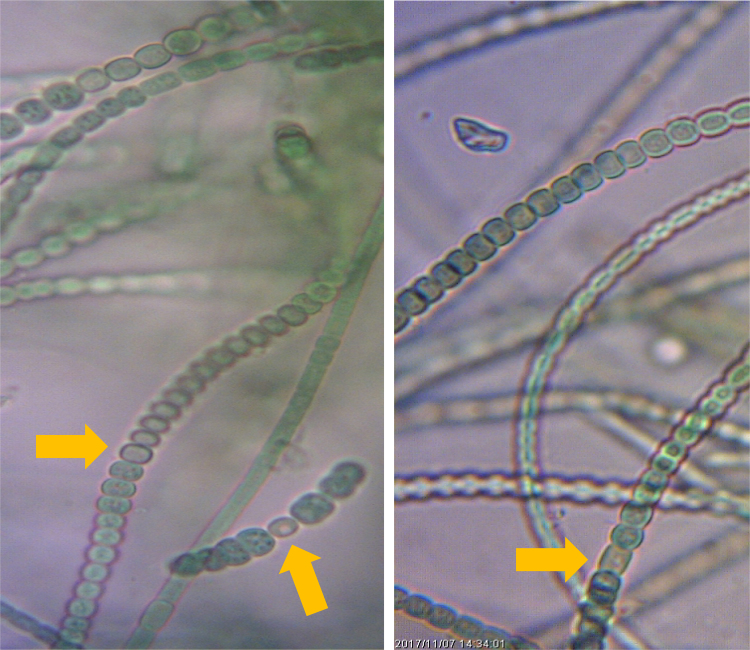
Heterocysts in vanadium (V+ Mo-) [Left] and in molybdenum (Mo+ V-) cultures of *Fischerella uthpalarensis* (pointed as orange arrows).

**Fig. 3:**
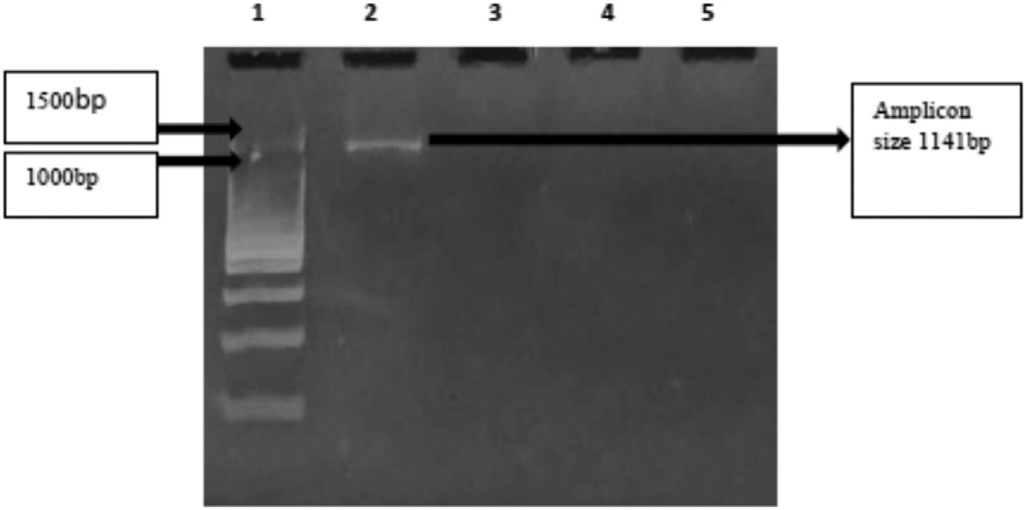
PCR products of *vnfDG* gene for *Nostoc azollae* (Major cyanobiont) and the cultured free-living cyanobiont (*Fischerella uthpalarensis*) of *A. pinnata* amplified using VnfDG1F & VnfDG6R primer pair. Lane 1- DNA ladder (100 bp); Lane 2- PCR product of the positive control; Lane 3- No PCR product of the negative control; Lane 4 and Lane 5- No PCR products for genomic DNA templates from *Nostoc azollae* and the cultured free living cyanobiont.

Our culture data, which show decisively and conclusively, that there is significant growth inside vanadium cultures (V+ Mo-), just as much, or better than molybdenum cultures (Mo+ V-), pointing to a dual nitrogenase system inside *Fischerella uthpalarensis*, a minor cyanobiont isolated from *Azolla pinnata* (Fig. 1). Furthermore there is no growth in cultures absent in both vanadium and molybdenum (Fig. 1). Although we observed heterocysts in vanadium (V+ Mo) cultures (Fig. 2), compartments where nitrogen fixation takes place, we were unable to mine the genetic determinants of this possible vanadium-dependent nitrogen fixation (Fig. 3). The vanadium dependency of the Azolla cyanobiont *F. uthpalarensis*, could be used in multiple pragmatic facets, namely in brief 1. To quench vanadium using nitrogen fixation 2. To use *Azolla pinnata*, as a biofertilizer, that will run on a strong vanadium dependent nitrogenase enzyme 3. To promote the synthesis of cadmium sequestering proteins such as metallothioneins, by vanadium-dependent nitrogen fixation, to remediate cadmium, in CKDu prevalent areas (due to CKDu areas harboring high bioavailable vanadium).

## 2 Experimental design, materials and methods

### 2.1 Plant material

The water fern *Azolla pinnata* was obtained from the Rice Research and Development Institute (RRDI) and was authenticated as *Azolla pinnata* R. Brown of family Azollaceae by the National Herbarium, Peradeniya, Sri Lanka.

### 2.2 Isolation and cultivation of the culturable cyanobiont of *Azolla pinnata* R. Brown

The ferns were grown in a tank with 2 L of distilled water supplied with 1 g L^−1^Albert solution (10.6% N, 9.3% P, 16.3% K, 11% Ca, 2.25% Mg, 35 mg/kg B, 35 mg/kg Cu, 660 mg/kg Fe, 130 mg/kg Mn, 140 mg/kg Zn and 20 mg/kg Mo) [5] for acclimatization and to achieve a higher biomass. For each isolation process, 15 g of fresh weight of fern tissues were taken washed thoroughly in running tap water for 10 min and surface disinfected. A robust disinfection consisting of 2 min rinse, in 10% Clorox, and 1 min rinse in sterile 0.01 M HCL was employed. After the disinfection, ferns were given two 1 min rinses in sterile distilled water.

The disinfected ferns were crushed and homogenized in 50 mL of one-eighth-strength N-free BG11_0_(BG11_0_/8) [6], filtered and centrifuged for 5 min at 500 *g* (HERMLE, Z206A, Wehingen, Germany). The resulting cell pellet was washed in 2 mL of BG11_0_/8 and 500 μL was transferred to fresh 10 mL of BG11_0_/8 in a sterile McCartney bottle. The McCartney bottle was incubated with intermittent hand shaking for two weeks under natural light conditions (approximately 12 h day and night cycle) and temperature prevailing in Colombo, Sri Lanka. However, it was not exposed to direct sun light but kept near a closed un-tinted window in the laboratory to receive sunlight during the day. Following incubation, 5 mL of the medium was transferred to fresh 10 mL BG11_0_/8 and incubated for another two weeks under same conditions as previously described. At the end of 2 weeks, a higher volume from the medium (10 mL), was transferred again to a higher volume of fresh BG11_0_/8 medium (20 mL) in a conical flask and incubated on a gyrotory shaker at 150 rpm (Digital orbital shaker, TS-560D, China) under same light conditions and temperature. Following 1 week of incubation, the low strength medium harboring cyanobionts was supplied with full strength BG11_0._ An aliquot of 3 mL was supplied in every five days until a higher bio mass was apparent.

This protocol was replicated three times (3 technical replicates) to assess its reproducibility and the effectiveness in isolating and cultivation of a culturablecyanobiont.

The culture composition of N-free BG11_0_ with Molybdenum (without Vanadium) (V-Mo+) is given below.

**Composition of N-free BG11**_**0**_ **with Molybdenum (without Vanadium) (V-Mo+)**

**Table:** 

NaCl	1.5 g
K_2_HPO_4_	0.04 g
MgSO_4_·7H_2_O	0.075 g
CaCl_2_·2H_2_O	0.036 g
Citric acid	0.006 g
Ferric citrate	0.006 g
EDTA (disodium salt)	0.001 g
Na_2_CO_3_	10 g
Trace metal mix	1 mL
Distilled water	1.0 L

Adjust the PH to 7.1.

**Trace metal mix**

**Table:** 

H_3_BO_3_	2.86 g
MnCl_2_·4H_2_O	1.81 g
ZnSO_4_·7H_2_O	0.222 g
NaMoO_4_·2H_2_O	0.39 g
CuSO_4_·5H_2_O	0.079 g
COCl_2_·6H_2_O	49.4 mg
Distilled water	1.0 L

### 2.3 Determination of the growth of isolated free living cyanobiont in N-free molybdenum deficient, vanadium containing BG11o medium

In this experiment three types of media were used:

1. N-free BG11_0_ without molybdenum or vanadium (Mo-V-): **Negative control**.
2. N-free BG11_0_ with molybdenum devoid of vanadium (molybdenum only) (Mo+V-): **Positive control**.
3. N-free BG11_0_ with vanadium devoid of molybdenum (vanadium only) (Mo-V+): **Test**.

First, the isolated free living cyanobiont was grown in “Mo-V-” for two weeks to deplete the internal pools of metal ions. The “Mo-V+” medium was prepared using the same culture composition given by Ripka et al. [6], but the concentration of molybdenum was substituted with the same concentration of vanadium using NaVO_3_ salt. As the isolated cyanobiont formed clumps, an equal concentration of the species could not be determined using spectroscopic measurements. Therefore, an approximately equal amount of the cyanobionts (1 mL of the medium containing the cyanobionts) was transferred from “Mo-V-” to three different 250 mL flasks containing 100 mL of Mo-V+, Mo+V-, Mo-V- and incubated for 4 weeks.The incubation conditions used were same as used during the isolation and culturing of the cyanobiont.

#### 2.3.1 Microscopic observations of the cultured cyanobiont

Four weeks old culture, was taken in to a sterile falcon tube and was crushed using a sterile pipette tip. After that wet mounts were prepared and were observed under the microscope (Dinolite digital microscope, Torrance, California, USA).

### 2.4 DNA extraction and characterization

DNA extraction was carried out by modifying a protocol described in current protocols in molecular biology [7]. DNA extraction was performed on the free living cyanobiont, the positive (*Azotobacter vinelandii*) and negative control *(E. coli)*.

### 2.5 Selection of vnf primers

PCR was done using the primer sequences given by [8]. To amplify fragments of vnfDG, and vnfN, primers VnfDG1F and vnfDG6R & VnfN2F and VnfN4R were used. Using the primer map below, VnfDG1F forward primer was combined with VnfDG6R reverse primer. Similarly, to amplify the vnfN fragments, VnfN2F forward primer was combined with VnfN4R reverse primer.

**Figure:**
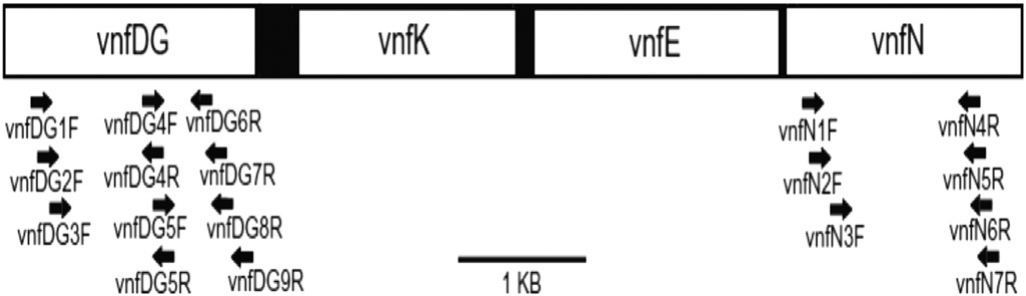

The above primer map of the vnf gene cluster in cyanobacteria has been portrayed, as appeared in Hodkinson et al., 2014 [8].

**Table:** 

**Primers and their sequences used in the amplification of*****vnf*****genes**	Sequence (5׳–3׳)
vnfDG1F	TATTAAAGTGCGACGAAAC
VnfDG6R	CCATCATCAATATAGAT
VnfN2F	AAAGATGTCAGTATTGT
VnfN4R	GCCATGTATTTTTCCCA

### 2.6 Optimized PCR protocol of *vnf* gene primers

The selected vnf primers were optimized using temperature gradient PCR (BIO-RAD, Berkeley, California, USA). From the combined primer pairs VnfDG1F & VnfDG6R and VnfN2F &VnfN4R, only VnfDG1F and VnfDG6R gave bands with *A. vinelandii* (positive control). The best annealing temperature of the primer pair VnfDG1F and VnfDG6R was found as 43.4 °C.

The PCR reaction mixture was prepared using template DNA, 10× PCR buffer (SIGMA-ALDRICH, containing 15 mM Mgcl2), dNTPs, Forward and reverse primers, Taq DNA polymerase and sterile nuclease free water as given in the table below.

**Table:** 

**Composition of 25 μL of PCR master mixture chemical ingredient**	**Amount added for 25 μL PCR reaction (μL)**
10× PCR buffer	2.5
200 μM dNTPs	1.0
Forward primer (10 μM/μL)	1.0
Reverse primer (10 μM/μL)	1.0
5 units/μL*Taq*DNA polymerase	0.3
Sterile Nuclease free water	16.2
DNA template	3.0

Each PCR amplification consisted of an initial denaturation step at 94 °C for 1 min followed by a process of 35 cycles consisted of three steps namely, a denaturation step at 94 °C for 30 s, annealing step at 43.4 °C for 30 s and extension step at 72 °C for 2 min. At the end of the final cycle, final extension was carried out at a temperature of 72 °C for 10 min, with subsequent holding temperature at 4 °C. Amplified PCR products were analyzed using 1.5% agarose gel electrophoresis. The size of the band was confirmed using 100 bp ladder (Promega, Madison, Wisconsin, USA).
